# Diverse Subclade Differentiation Attributed to the Ubiquity of *Prochlorococcus* High-Light-Adapted Clade II

**DOI:** 10.1128/mbio.03027-21

**Published:** 2022-03-14

**Authors:** Wei Yan, Xuejin Feng, Ta-Hui Lin, Xingyu Huang, Le Xie, Shuzhen Wei, Kun Zhou, Yi-Lung Chen, Weicheng Luo, Wenqian Xu, Wei Zhang, Muhammad Zohaib Nawaz, Ya-Wei Luo, Qinglu Zeng, Rui Zhang, Nianzhi Jiao

**Affiliations:** a College of Marine Science and Technology, China University of Geosciences, Wuhan, People’s Republic of China; b State Key Laboratory of Marine Environmental Science, Fujian Key Laboratory of Marine Carbon Sequestration, College of Ocean and Earth Sciences, Xiamen University, Xiamen, People’s Republic of China; c Shenzhen University-HKUST Joint Marine Science PhD Program, Shenzhen University, Shenzhen, People’s Republic of China; d Department of Ocean Science, Hong Kong University of Science and Technology, Kowloon, Hong Kong SAR, People’s Republic of China; e Department of Statistics, China University of Geosciences, Wuhan, People’s Republic of China; f Division of Life Science, Hong Kong University of Science and Technology, Kowloon, Hong Kong SAR, People’s Republic of China; g Hong Kong Branch of Southern Marine Science and Engineering Guangdong Laboratory (Guangzhou), Hong Kong University of Science and Technology, Hong Kong SAR, People’s Republic of China; Oregon State University

**Keywords:** *Prochlorococcus* HLII, subclade differentiation, pangenome, metagenome

## Abstract

*Prochlorococcus* is the key primary producer in marine ecosystems, and the high-light-adapted clade II (HLII) is the most abundant ecotype. However, the genomic and ecological basis of *Prochlorococcus* HLII in the marine environment has remained elusive. Here, we show that the ecologically coherent subclade differentiation of HLII corresponds to genomic and ecological characteristics on the basis of analyses of 31 different strains of HLII, including 12 novel isolates. Different subclades of HLII with different core and accessory genes were identified, and their distribution in the marine environment was explored using the TARA Oceans metagenome database. Three major subclade groups were identified, *viz.*, the surface group (HLII-SG), the transition group (HLII-TG), and the deep group (HLII-DG). These subclade groups showed different temperature ranges and optima for distribution. In regression analyses, temperature and nutrient availability were identified as key factors affecting the distribution of HLII subclades. A 35% increase in the relative abundance of HLII-SG by the end of the 21st century was predicted under the Representative Concentration Pathway 8.5 scenario. Our results show that the ubiquity and distribution of *Prochlorococcus* HLII in the marine environment are associated with the differentiation of diverse subclades. These findings provide insights into the large-scale shifts in the *Prochlorococcus* community in response to future climate change.

## INTRODUCTION

The marine cyanobacterium *Prochlorococcus* is the most abundant and smallest known oxygenic photosynthetic microorganism, and it is mainly distributed in the euphotic zone of tropical and subtropical oligotrophic oceans. Its abundance in marine environments typically ranges from 10^4^ to 10^5^ cells mL^−1^, which accounts for 30% to 80% of the total photosynthetic biomass and net primary productivity in certain regions of the open ocean (1–8). According to recent estimates, the annual mean global abundance of *Prochlorococcus* in the ocean is approximately 3 × 10^27^ cells, and this value is expected to increase by one-third by the end of the 21st century in response to global warming under the Representative Concentration Pathway (RCP) 4.5 scenario (5).

Based on physiological characteristics, ecological distribution, and phylogeny, the 12 *Prochlorococcus* clades can generally be divided into two ecotypes: the high-light-adapted (HL) clades and low-light-adapted (LL) clades (9–20). High-light-adapted clade II (HLII) and its counterpart, HLI, which are distinguished by their optimal growth temperature (17), have a key evolutionary feature known as genome streamlining, which helps these cyanobacteria to reproduce rapidly under energy-limiting and nutrient-starved conditions in oligotrophic oceans (4, 21, 22). HLII account for more than 90% of total *Prochlorococcus* in tropical open oceans (17). Thus, bearing in mind the huge abundance of *Prochlorococcus*, HLII is one of the most abundant photosynthetic organisms on the planet if we consider it as a distinct cyanobacterial species.

The highly compact genome of individual HLII strains restricts their ability to adapt to a wide range of environmental conditions. Because HLII populations in the euphotic zone are continuously exposed to steep gradients of environmental factors, such as light intensity, temperature, and nutrient concentrations, they have evolved to respond and adapt to such conditions (20, 22–25). Additionally, the extremely large populations and relatively high growth rates of HL *Prochlorococcus* promote differentiation among closely related strains and impose strong selective pressures within the ecological niches in surface and subsurface waters (22). Several studies have reported a strong relationship between HLII gene content and environmental variables (26–28). Two environmental single-cell genomic studies revealed that subpopulations tend to coexist within HLII (23, 24). Investigations based on marker genes, including environmental ITS (16S–23S rRNA internal transcribed spacer) (20, 25), *petB* (29), and *rpoC1* sequences (30–32), revealed associations between HL subpopulations and environmental parameters. Overall, the results of those studies revealed the existence of diverse ecologically coherent subclades within HLII and identified potentially disparate properties that were previously masked by coarse phylogenetic resolution.

The genomic and physiological differences among HLII subclades and the influence of ecological niches on subclade differentiation remain largely unexplored, mainly due to the inadequate number of available whole genomes for detailed mining and the lack of large-scale environmental metagenomic studies. This gap, therefore, hampers our understanding of how HLII became the most abundant *Prochlorococcus* clade. In this study, we isolated 12 novel strains of *Prochlorococcus* HLII from the western Pacific Ocean and the South China Sea and performed a genomic analysis of 31 different *Prochlorococcus* HLII genomes. The environmental occurrence of HLII subclades was explored using data from the TARA Oceans metagenomic database (33). Finally, the environmental factors that drive HLII subclade distribution were identified using regression models, and their shifts in relative abundances in response to future global warming were predicted.

## RESULTS

### New HLII isolates from the western Pacific Ocean.

**FIG 1:**
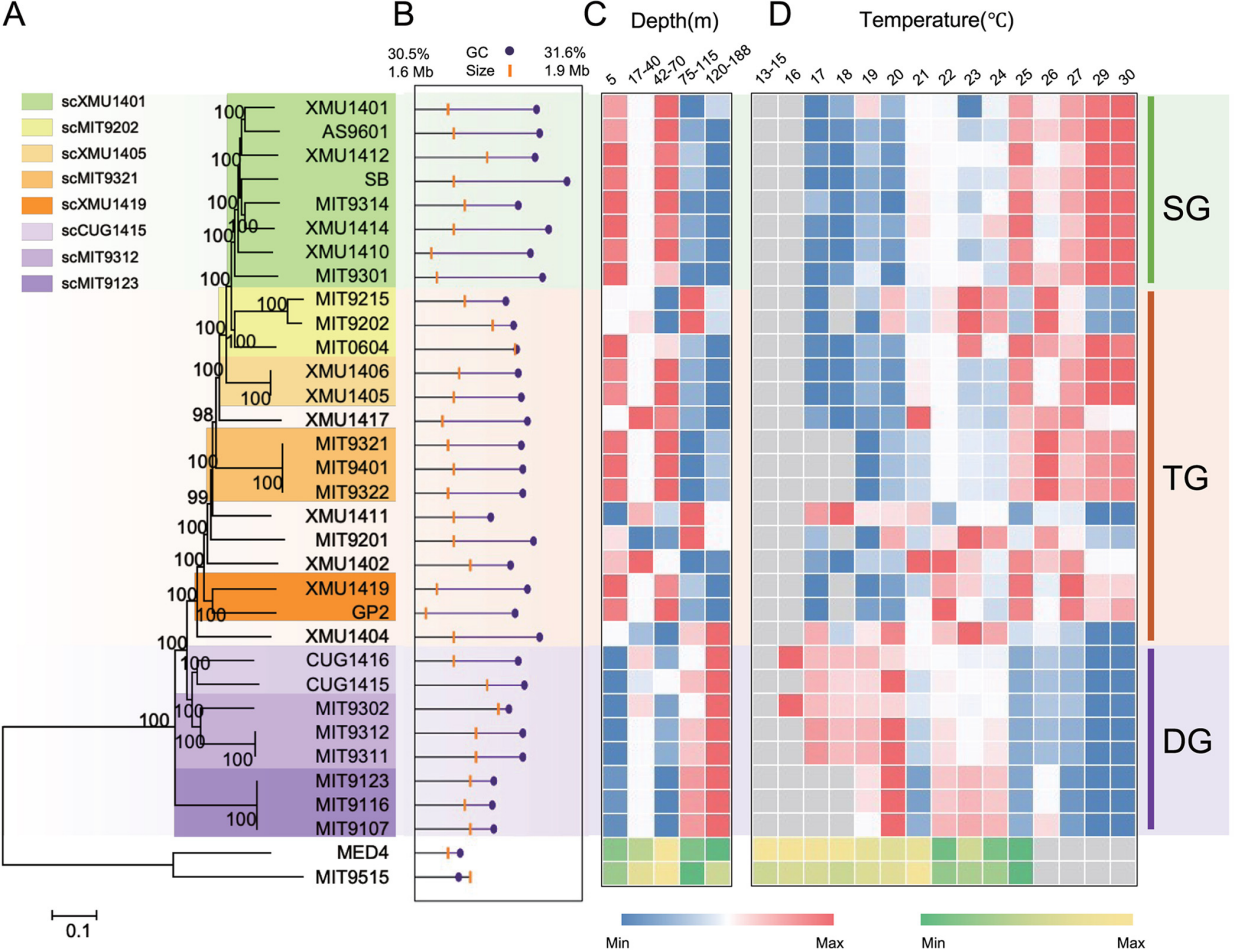
Phylogenomic diversity and environmental distribution of HLII strains. (A) Maximum likelihood phylogenetic tree constructed from the core genome single nucleotide polymorphisms (SNPs) of 31 *Prochlorococcus* HLII strains, with two HLI strains as an outgroup. Numbers at nodes are bootstrap values for 1000 resamplings (at least 80% support). (B) Genome characteristics and isolation depths of 33 HL strains. Heatmaps of log-transformed relative abundances in TARA Oceans metagenome libraries for each strain at different depths (C) and temperatures (D). Color gradient indicates increasing values of the relevant measurement from minimums (blue or green) to maximums within a row (red or yellow). Gray color indicates no detection. SG, TG, and DG indicate *Prochlorococcus* HLII surface group, transition group, and deep group, respectively. Relative abundances of HLII strains were normalized to the total abundance of all HLII reads. As an outgroup, relative abundances of HLI strains were normalized to the total abundance of HLI and HLII reads.

Twelve new *Prochlorococcus* strains were isolated from depths of 50 to 300 m from six different sites in the western Pacific Ocean and the South China Sea (see Table S1 and Fig. S1). Only one ITS sequence was found in the contigs belonging to each genome, which confirmed the unialgal status of the HLII cultures. Their taxonomic positions were confirmed through comprehensive phylogenetic analyses (see below and Fig. 1A and Fig. S2 and S3). The genome size of the 12 HLII strains ranged from 1,631,014 bp to 1,734,399 bp, with an average GC content of 31.38% (standard deviations [SD] of 0.09) and an average of 1,964 (SD of 37) coding sequences per genome (Fig. 1B and Table S1). Two strains, including CUG1417 and XMU1419, were isolated from a depth of 300 m, well below the euphotic zone and near the Luzon Strait in the South China Sea. These two strains represent *Prochlorococcus* of the deepest origin to date.

10.1128/mbio.03027-21.1 FIG S1 (A) Isolation locations of the 12 *Prochlorococcus* HLII strains included in the current study. (B) Sampling locations of the 107 TARA Oceans metagenomes included in the current study. Circled dot indicates more than one sampling depth in a TARA Oceans station. Download FIG S1, PDF file, 0.4 MB. Copyright © 2022 Yan et al. 2022 Yan et al. https://creativecommons.org/licenses/by/4.0/ This content is distributed under the terms of the Creative Commons Attribution 4.0 International license.

10.1128/mbio.03027-21.2 FIG S2 Phylogenomic relationships of *Prochlorococcu*s. Phylogenetic trees of HL *Prochlorococcu*s strains reconstructed by ITS (A) and concatenated protein sequences of 31 core genes (B) with RAxML v8.0. Two HLI strains (MIT9515 and MED4) were used as an outgroup. Phylogenetic trees of HL and LL *Prochlorococcu*s strains reconstructed by ITS (C) and concatenated protein sequences of 31 core genes (D) with RAxML v8.0. *Synechococcus* WH5701 was used as an outgroup. Bootstrap values for 1,000 resamplings are indicated by numbers at the nodes (at least 50% support). Download FIG S2, PDF file, 0.4 MB. Copyright © 2022 Yan et al. 2022 Yan et al. https://creativecommons.org/licenses/by/4.0/ This content is distributed under the terms of the Creative Commons Attribution 4.0 International license.

10.1128/mbio.03027-21.3 FIG S3 Phylogenomic relationships of *Prochlorococcus* HLII strains. Phylogenetic trees (A to F) were reconstructed using concatenated protein sequences of core genes defined at multiple similarity cutoffs from 50 to 95% with RAxML v8.0. Phylogenetic trees (G to L) were reconstructed using concatenated SNPs of core genes defined at multiple similarity cutoffs from 50 to 95% with RAxML v8.0. Two HLI strains (MIT9515 and MED4) were used as an outgroup. Bootstrap values for 1,000 resamplings are indicated by numbers at the nodes (at least 50% support). Download FIG S3, PDF file, 0.4 MB. Copyright © 2022 Yan et al. 2022 Yan et al. https://creativecommons.org/licenses/by/4.0/ This content is distributed under the terms of the Creative Commons Attribution 4.0 International license.

10.1128/mbio.03027-21.7 TABLE S1 Isolation locations, genome characteristics, and assembly statistics for *Prochlorococcus* HLII strains used in this study. Download Table S1, XLSX file, 0.02 MB. Copyright © 2022 Yan et al. 2022 Yan et al. https://creativecommons.org/licenses/by/4.0/ This content is distributed under the terms of the Creative Commons Attribution 4.0 International license.

### Phylogeny of HLII subclades.

The phylogeny of the HLII subclades was inferred using ITS sequences and concatenated protein sequences from multiple core genes (defined at similarity cutoffs ranging from 50% to 95%). In most cases, the resulting phylogenetic trees were topologically similar and showed that *Prochlorococcus* HLII consists of several subclades (Fig. S2 and S3). Notably, the branch lengths of the core gene protein trees were relatively short, mainly because protein sequences encoded by core genes are highly conserved and all HLII strains are relatively closely related to each other (Fig. S3). Thus, to improve the resolution of the *Prochlorococcus* HLII subclade phylogeny, we constructed phylogenetic trees using core genome single nucleotide polymorphisms (SNPs) at a range of similarity cutoffs (50% to 95%) (Fig. 1A and Fig. S3). Figure 1A shows the core genome SNP tree (at similarity cutoff of 60%) with high bootstrap support for its topology. The SNPs were distributed throughout the genome with high abundance and genetic stability, whereas the core genome contained essential genes that are often vertically transmitted and have a strong signal-to-noise ratio for phylogenetic inference, resulting in a high phylogenetic resolution (34). Thus, the tree constructed on the basis of core genome SNPs represented the clear phylogenetic relationships among *Prochlorococcus* HLII genomes and suggests that the 31 HLII strains could be divided into several subclades, including scXMU1401, scMIT9202, scXMU1405, scMIT9321, scXMU1419, scCUG1415, scMIT9312, and scMIT9123 (Fig. 1A).

### Environmental occurrences of HLII subclades.

To estimate the relative abundance and distribution patterns of the HLII subclades in marine environments, their genomes were mapped to 107 TARA Oceans metagenomic samples (0.2- to 1.6-μm- and 0.2- to 3-μm-size fractions) covering the surface/subsurface (5 to 188 m) layers of temperate to tropical oceans (Fig. S1 and Table S2). The results show that HL (including HLI and HLII) genomes were detected in 89 metagenomes, and the HLII subclades differed in their relative abundance at different depths and temperatures. Three ecological subclade groups of *Prochlorococcus* HLII strains, *viz.*, the surface group (HLII-SG), the transition group (HLII-TG), and the deep group (HLII-DG), were defined based on their relative abundances at various depths initially, which interestingly showed an obvious pattern of temperature (Fig. 1D). The strains belonging to basal clades (highlighted with violet) demonstrated the maximum relative abundance of representatives dwelling in deep waters with a continuous and obvious pattern (Fig. 1C) and were regarded as HLII-DG. Similarly, representative strains of the terminal clade (shown in green) demonstrate a maximum abundance of strains inhabiting the surface water and were regarded as HLII-SG (Fig. 1C). Noticeably, HLII-SG mainly represents the high-temperature strains, whereas low-temperature strains mainly constitute the HLII-DG (Fig. 1C and D and Fig. S4). The strains representing the middle clades and showing dispersal patterns were named HLII-TG. HLII-SG, containing mainly scXMU1401, accounted for more than 52% of all HLII reads in our data set after normalizing the sizes of all libraries (Fig. S4). HLII-SG was most abundant in the upper euphotic layer (5 to 70 m) (Fig. 1C) and showed the highest relative abundance within the temperature range of 25°C to 30°C (Fig. 1D). In contrast, the HLII-DG group (scCUG1415, scMIT9312, and scMIT9123) was mainly restricted to mid-deep layers (75 to 188 m) (Fig. 1C) and had a lower temperature range of 16°C to 23°C (Fig. 1D). Notably, scMIT9123 was distantly related to all other HLII subclades in the phylogenetic tree. The strains in this subclade were isolated from a depth of 25 m (Table S1). However, analyses of its overall distribution pattern indicated that this subclade inhabits mid- or deep layers (Fig. 1C); therefore, it was grouped into HLII-DG. The scMIT9202, scXMU1405, scMIT9321, and scXMU1419 subclades in HLII-TG showed a complex distribution pattern with respect to depth and temperature. For example, HLII-TG subclades, including scXMU1405 and scMIT9321, demonstrated distribution patterns closer to that of HLII-SG, whereas the HLII-TG subclades, including scMIT9202 and scXMU1419, showed distribution patterns similar to that of HLII-DG (Fig. 1C and D). The outgroup, consisting of HLI strains, was dominant in the temperature range of 13°C to 23°C, in contrast to the temperature distribution patterns of the HLII subclades (Fig. 1D). Because depth is negatively correlated with light intensity in the water column, these observed distribution patterns reflect the significance of both light and temperature in shaping the distribution of the ecologically coherent subclades of HLII in the ocean.

10.1128/mbio.03027-21.4 FIG S4 Phylogenomic diversity and environmental distribution of HLII strains. (A) Maximum likelihood phylogenetic tree reconstructed from the core genome single-nucleotide polymorphisms (SNPs) of 31 *Prochlorococcus* HLII strains, with two HLI strains as an outgroup. Numbers at nodes are bootstrap values for 1,000 resamplings (at least 80% support). Heatmaps of log-transformed relative abundances in TARA Oceans metagenome libraries for each strain at different depths (B) and temperatures (C). Relative abundances of HLII strains were normalized to the total abundance of all HLII reads. As an outgroup, relative abundances of HLI strains were normalized to the total abundance of HLI and HLII reads. Color gradient indicates increasing values of log-transformed relative abundances. Gray color indicates no detection. SG, TG, and DG indicate *Prochlorococcus* HLII surface group, transition group, and deep group, respectively. (D) Current overall relative abundance of the *Prochlorococcus* HLII subclades and the HLI clade in 89 TARA Oceans metagenomes (defined as the proportion of reads recruited to each genome relative to the total number of reads recruited to the entire library of 31 HL and 3 HLI genomes). Download FIG S4, PDF file, 0.4 MB. Copyright © 2022 Yan et al. 2022 Yan et al. https://creativecommons.org/licenses/by/4.0/ This content is distributed under the terms of the Creative Commons Attribution 4.0 International license.

10.1128/mbio.03027-21.8 TABLE S2 TARA Oceans sample description and associated metadata. Download Table S2, XLSX file, 0.04 MB. Copyright © 2022 Yan et al. 2022 Yan et al. https://creativecommons.org/licenses/by/4.0/ This content is distributed under the terms of the Creative Commons Attribution 4.0 International license.

### Differentiation of the HLII core genome.

**FIG 2:**
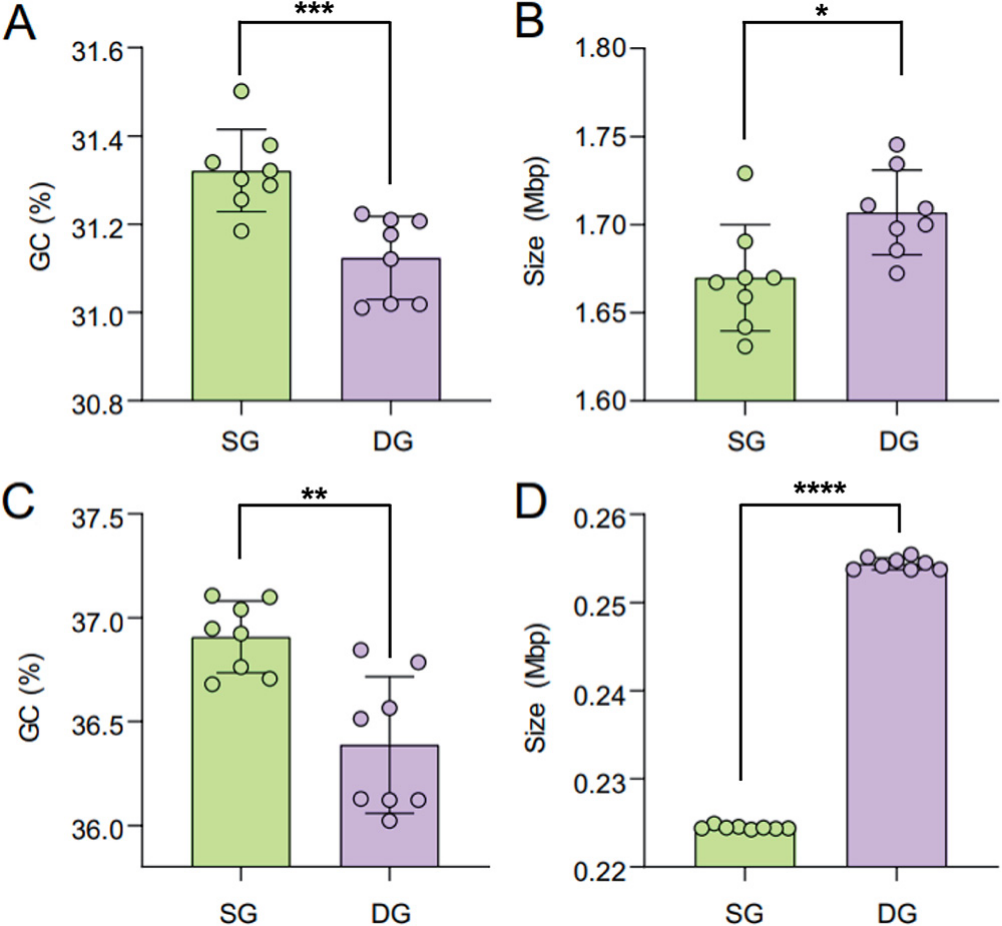
Contrasting *Prochlorococcus* HLII surface group and deep group. Panels A and B represent genome sizes and GC contents of whole genomes from *Prochlorococcus* HLII surface group (HLII-SG) and deep group (HLII-DG), respectively. Panels C and D represent genome sizes and GC contents of core genome single nucleotide polymorphisms (SNPs) from HLII-SG and HLII-DG, respectively. Colored circles represent individual genome values, and bars indicate one standard deviation. The number of colored circles denotes the number of statistical tests showing a significant difference (*, *P* < 0.05; **, *P* < 0.01; ***, *P* < 0.001; ****, *P* < 0.0001).

On the whole-genome scale, we observed differences in GC content and genome size between HLII-SG and HLII-DG. The average GC content of HLII-SG (31.32% ± 0.09%) was higher than that of HLII-DG (31.12% ± 0.09%) (*P = *0.0008, two-sample *t* test, *n *= 8) (Fig. 2A), and the average genome size of HLII-SG (1.670 ± 0.028) was smaller than that of HLII-DG (1.707 ± 0.023 Mbp) (*P = *0.0164, two-sample *t* test, *n *= 8) (Fig. 2B). Additionally, the GC content of the core genome SNPs was higher in HLII-SG (36.91% ± 0.16%) than in HLII-DG (36.39% ± 0.31%) (*P = *0.0014, two sample *t* test, *n *= 8) (Fig. 2C). There were fewer core genome SNPs in HLII-SG (224,472 ± 190 bp) than in HLII-DG (254,402 ± 631 bp) (*P < *0.0001, two sample *t* test, *n *= 8) (Fig. 2D). These differences in GC contents are similar to those reported in previous studies that detected variations in GC contents among different *Synechococcus* and *Prochlorococcus* clades and subclades in different ecological niches, especially thermal and variable salinity niches (35, 36).

**FIG 3:**
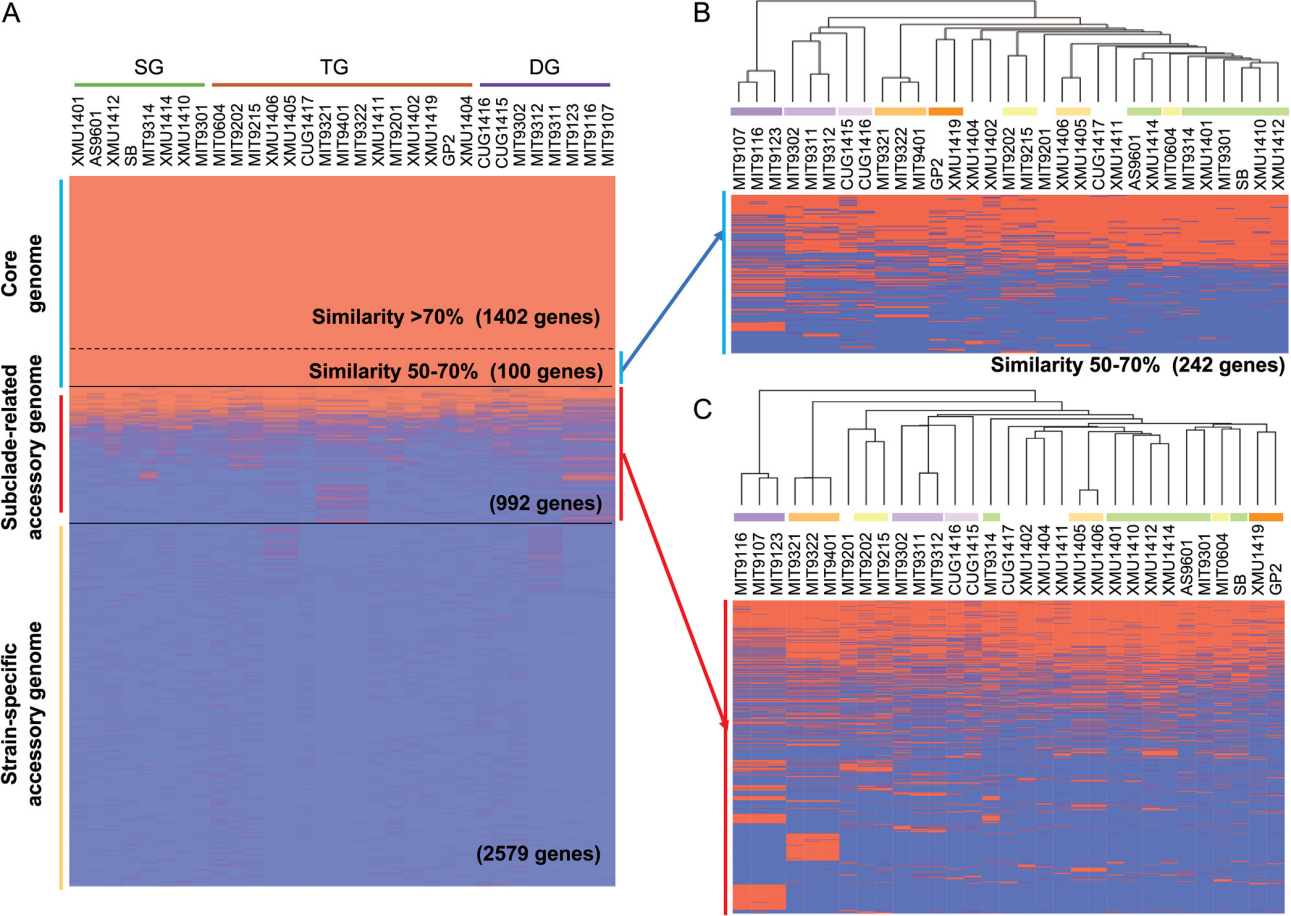
Pangenome of *Prochlorococcus* HLII. (A) Distribution of 5,073 genes in the pangenome of *Prochlorococcus* HLII. (B) Distribution of the genes which were considered core genes based on 50% amino acid similarity cutoff but fell in the category of accessory genes while using 70% cutoff. (C) Distribution of 992 accessory genes in the 31 genomes of *Prochlorococcus* HLII. Dendrograms above the two heatmaps show hierarchical clustering of the 31 HLII genomes based on gene distribution (Euclidian distance). SG, TG, and DG indicate *Prochlorococcus* HLII surface group, transition group, and deep group, respectively.

In this study, the HLII pangenome was separated into three parts: the core genome (genome fragments/genes shared by all 31 HLII genomes), the accessory genome (genome fragments/genes shared by 3 to 30 HLII genomes, thereby targeting most genes potentially associated with subclade differentiation), and the strain-specific accessory genome (genome fragments that appeared in only one or two strains) (Fig. 3A). Noticeably, in this study, the pangenome analysis was performed among strains of the same ecotype, which are closely related to each other. For example, strains MIT9107, MIT9116, MIT9321, and MIT9401 share average nucleotide identity of >99% with MIT9116, MIT9123, MIT9322, and MIT9321, respectively. Considering such nearly identical genomes, genes limited to one/two strains were regarded as strain-specific genes and excluded from accessory gene analysis, since we tried to target most genes that might be related to subclade differentiation.

To identify core genes associated with subclade differentiation, we applied two similarity cutoffs to the 31 HLII genomes: a 50% similarity cutoff (amino acid level) resulted in 1,502 core genes, and a 70% cutoff resulted in 1,402 core genes (Fig. 3A). Therefore, 100 core genes with amino acid similarity between 50% and 70% were identified. These genes were considered core genes based on 50% amino acid similarity cutoff but fell in the category of accessory genes (resulting in 242 genes) while using a 70% cutoff (Fig. 3B). Hierarchical clustering based on Euclidean distance was performed using the binary matrix of these 242 genes (Fig. 3B), and the resulting dendrogram was generally congruent with the core genome SNP tree (Fig. 1A). Several of these genes with known functions were related to high-light protection, DNA repair, and nutrient transport (Table S3). For example, the urea assimilation gene cluster that is present in most of the HLII genomes showed differences between HLII-SG and HLII-DG (Table S3).

10.1128/mbio.03027-21.9 TABLE S3 Table for genome analysis of HLII strains. Download Table S3, XLSX file, 0.09 MB. Copyright © 2022 Yan et al. 2022 Yan et al. https://creativecommons.org/licenses/by/4.0/ This content is distributed under the terms of the Creative Commons Attribution 4.0 International license.

At the SNP level, we used two filters (see Materials and Methods) to screen the 31 HLII core genomes. We found that most of the regions with substantial SNP variations were located between or within genes with unknown function (Fig. S5). Interestingly, three core genes (*chlB*, *nblS*, and *amtB*) were found to have relatively large SNP differences between HLII-SG and HLII-DG (Fig. S5). The *chlB* gene encodes a subunit of light-independent protochlorophyllide reductase, which is required for the light-independent accumulation of chlorophyll (37). The *nblS* gene encodes a protein that is involved in changes in the photosynthetic apparatus in response to both high light and nutrient limitation (38). The *amtB* gene encodes a low-affinity ammonium uptake protein that is a member of the Amt family of ammonium/ammonia transporters. The transporter is responsible for bidirectional ammonium diffusion across the membrane and is only necessary for growth at low ammonium concentrations (39, 40). These results suggest that the core genomes of HLII subclades have differentiated to adapt to the diverse environments in the stratified euphotic zone, e.g., to variations in light intensity and nutrient concentrations. A recent study also found that adaptation of the picocyanobacteria clade and subclades to ecological niches relies on amino acid substitutions in the core genome rather than on variations in gene content (35). Overall, our results show that HLII strains have a small, conserved core genome (1,402 genes, defined at 70% amino acid similarity), a subclade-specific core genome (100 genes defined at 50 to 70% amino acid similarity), and a subclade-specific accessory genome (992 genes) while having a large strain-specific genome (2,579 genes, although the actual size may be much larger).

10.1128/mbio.03027-21.5 FIG S5 (A) Visualization of core gene SNPs among different HLII subclades. The genome alignment was magnified to reveal the phylogenetic signatures (SNP) of the surface and deep groups using Parsnp. Two criteria were used to identify core genes with substantial SNP variation: (1) SNP in aligned 100-bp window with <50% volume identity or (2) SNP in aligned 100-bp window with >20 indels. Visualization of SNPs of three core genes, *chlB* (B), *nblS* (C), and *amtB* (D), among different HLII subclades. The genome alignment was zoomed to reveal the phylogenetic signatures (SNP) of the surface and deep groups using Parsnp. Download FIG S5, PDF file, 0.4 MB. Copyright © 2022 Yan et al. 2022 Yan et al. https://creativecommons.org/licenses/by/4.0/ This content is distributed under the terms of the Creative Commons Attribution 4.0 International license.

### Dynamics of evolution of HLII gene content.

**FIG 4:**
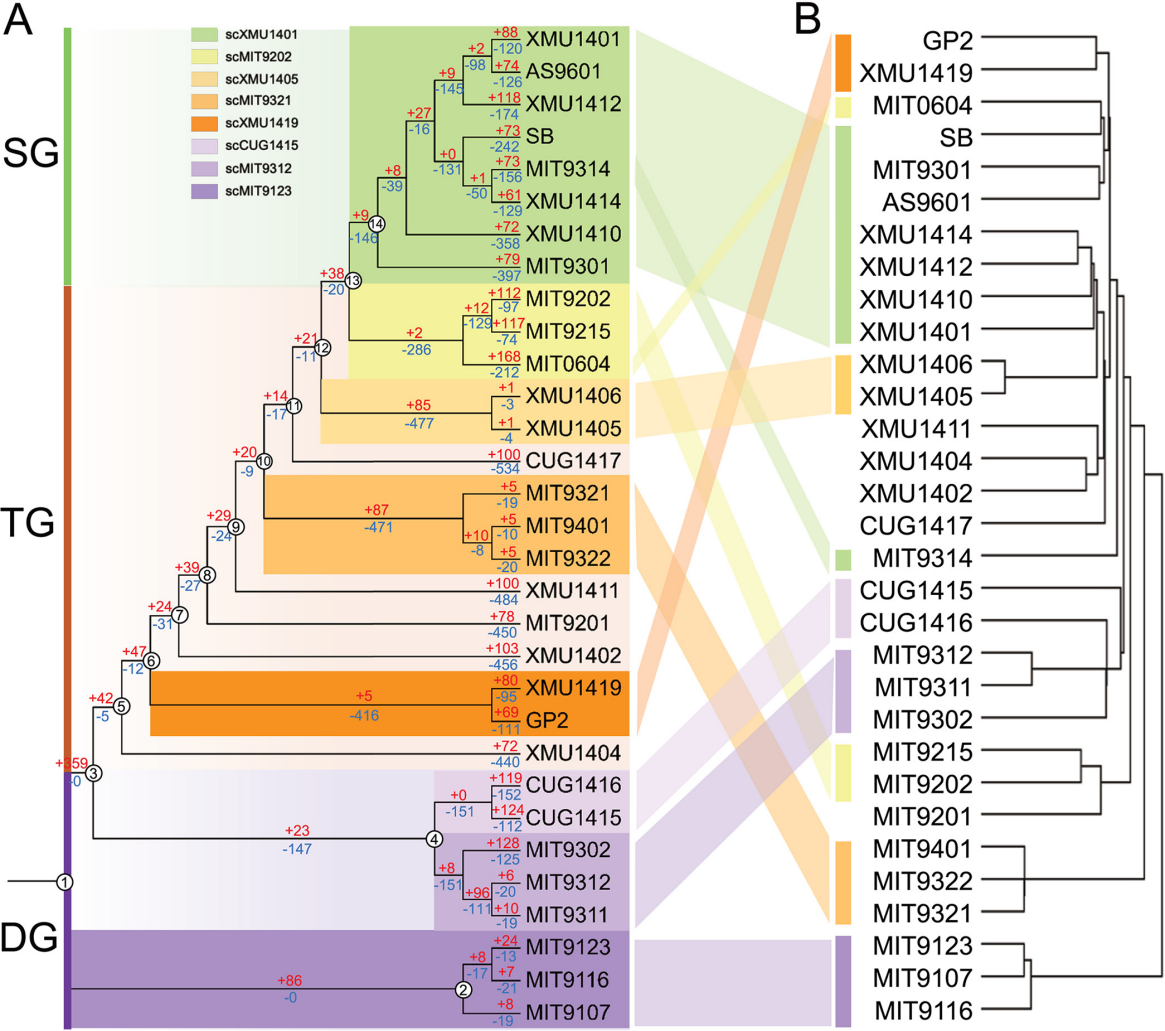
Relationships among *Prochlorococcus* HLII genomes based on phylogenomics (A) and on distribution (B) of 992 accessory genes. (A) Tree on the left shows the phylogeny constructed from core genome single-nucleotide polymorphisms (SNPs) of 31 *Prochlorococcus* HLII strains using maximum likelihood (same as Fig. 1A). Two HLI strains (MED4 and MIT9515) were used as an outgroup (not shown). Numbers on branches indicate estimated number of genes gained (red) and lost (blue) during evolution of *Prochlorococcus* HLII. (B) Tree on the right shows the clustering of the same HLII genomes based on the distribution of 992 accessory genes recovered from the pangenomic analysis (Euclidian distance).

The evolution of the HLII subclades was further explored based on their accessory genome content. In this analysis, the similarity between subclades was defined on the basis of their shared genes, and the underlying gene content was interpreted in terms of subclade ecological divergence. The topologies of the accessory genome tree and the core genome SNP tree were largely congruent (Fig. 4A and B [same as Fig. 3C]), indicating that accessory genome differentiation echoes core genome evolution. We found several genes in the accessory genome that were associated with ecological differentiation. For example, most genomes from HLII-DG lacked genes related to DNA repair that were present in HLII-SG genomes (e.g., DNA ligase, DEXH box helicase) (Table S3). Notably, all 31 HLII isolates carried *hli* genes encoding high-light-induced proteins (HLIPs) that are involved in protection against light-induced damage at the ocean surface (41). However, several *hli* genes were also found in the accessory genome, suggesting that different subclades carry different sets of *hli* genes (Table S3).

To get more insights into the evolutionary processes that led to the subclade differentiation of gene content within HLII genomes, we estimated the number of gene-gain and gene-loss events on each branch of the core genome SNP tree (Fig. 4A and Table S3). Branches 2 and 3, the first two branches after the divergence of the HLII ancestor (branch 1), gained 86 and 359 genes, respectively (Fig. 4A). Out of 359 genes gained on branch 3 in the core genome SNP tree, 15 were related to DNA replication and repair and 20 to transporters (Table S3). Seven genes were related to photosynthesis and light protection, three genes were related to nitrogen metabolism, and five genes were related to phosphorus metabolism (Table S3). Branches 2 and 3 exhibited large differences and were at the beginning of the evolutionary tree, indicating that scMIT9123 and the common ancestor of subclades other than scMIT9123 have been differentiated for a relatively long time after their divergence. Branch 3, containing the subclades other than scMIT9123, gained a large number of genes, indicating higher genetic diversity. This may have resulted from adaptation to the surface and subsurface ocean environments during evolutionary history. Branch 4, comprising HLII-DG in the deep euphotic zone, was subdivided into two distinct subclades, scMIT9312 and scCUG1415. One gene (*hli*) encoding HLIP has been lost from the strains positioned on branch 4 (Table S3), as they were not essential for adaptation in this environment due to its weak light intensity. Likewise, because the deeper layers of the euphotic zone have very little exposure to UV light, intracellular DNA is not damaged by UV radiation, and eight genes related to DNA repair functions have been lost from strains positioned on this branch.

In the core genome SNP tree, HLII-TG comprised a total of nine branches, from branches 5 to 13 (Fig. 4A) (Table S3). Overall, in this group, 14 transporter genes were acquired and nine were lost; nine DNA replication and repair genes were acquired and three were lost; two nitrogen metabolism genes were gained and three were lost; and one phosphorus metabolism gene was gained. Some HLII-TG subclades (including scXMU1419, scMIT9321, scXMU1405, and scMIT9202) might have been exposed to high light and high levels of UV radiation during the adaptation process in the upper and mid-euphotic zone. Therefore, constant acquisition of DNA repair-related genes was an evolutionary requirement to survive in this environment. Transporter genes were randomly gained and lost, which shows that adaption strategies evolved in response to fluctuating nutrient conditions in the upper and mid-euphotic layer during evolution in HLII-TG. Branch 14 lost 146 genes, the highest number lost among these branches (Fig. 4A) (Table S3), implying that the HLII-SG (scXMU1401) lost genes related to adaptation to the nutrient-poor environment of the surface layer and the genome was effectively streamlined to lower their nutrient and energy requirements.

### Drivers of global HLII subclade composition and distribution.

**FIG 5:**
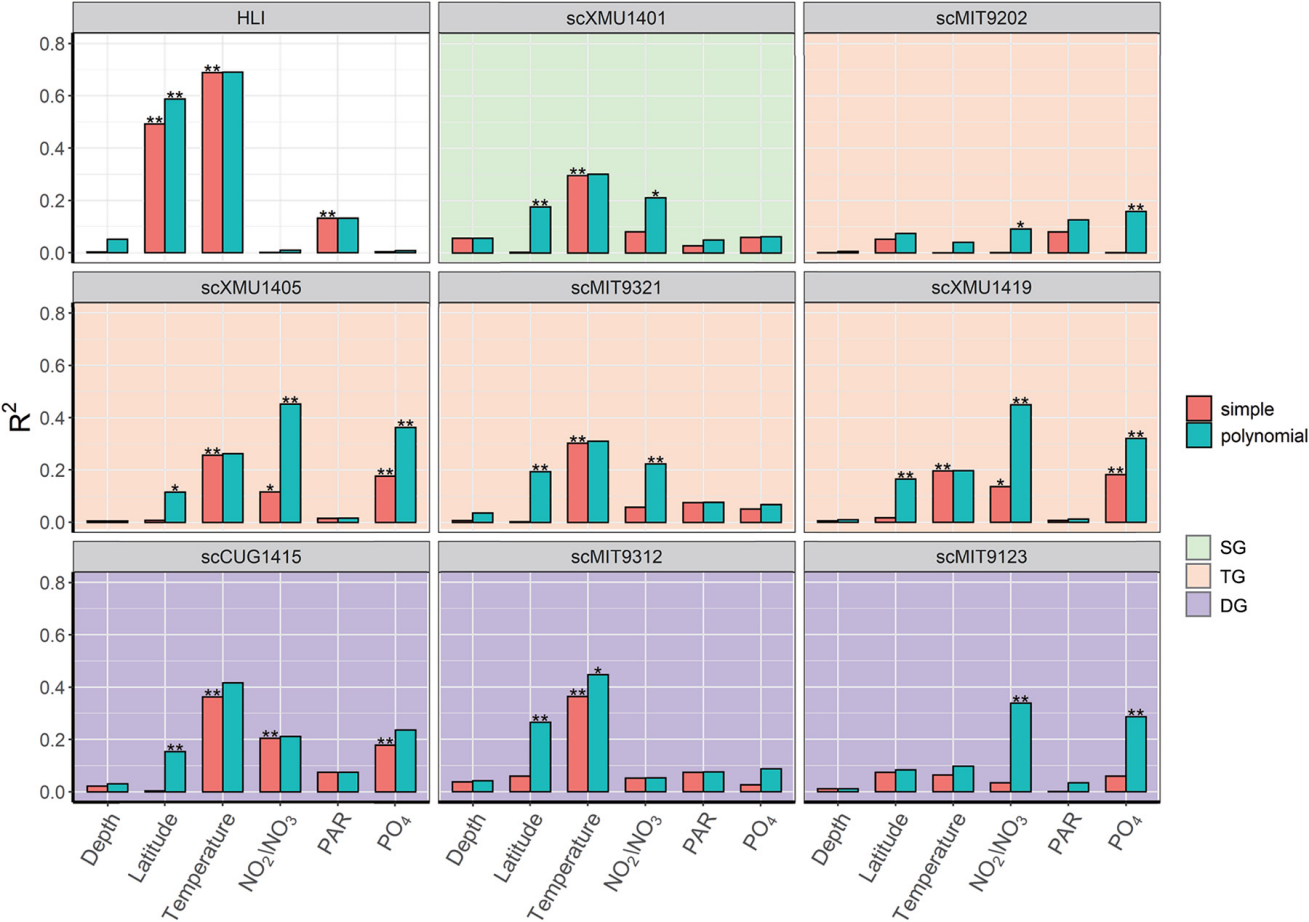
*R*-squared plots showing explanatory power of environmental variables for relative abundances of HLI and HLII subclades in univariate regression models. *, *P* < 0.05; **, *P* < 0.01. Red columns indicate linear models, and green columns indicate quadratic models. For statistical analyses, HLI relative abundance was normalized to all HL relative abundances (HLI and HLII). Relative abundances of HLII subclades were normalized to relative abundance of all HLII.

Potential relationships between the relative abundances of HLII subclades (normalized to the total abundance of all HLII reads) and environmental variables, including latitude, depth, temperature, and nutrient concentrations, were identified using univariate regression analyses (Fig. 5 and Table S4). Specifically, the relative abundance of scXMU1401 (HLII-SG) showed a positive relationship with temperature and a quadratic relationship with latitude and NO_2_/NO_3_ concentration. In HLII-DG, scCUG1415 showed relationships with temperature, latitude, and NO_2_/NO_3_ and PO_4_ concentrations. scMIT9312 showed relationships with latitude and temperature. scMIT9123 was affected by PO_4_ and NO_2_/NO_3_ concentrations. The subclades of HLII-TG showed complicated relationships with environmental drivers. All the subclades in this group were affected by NO_2_/NO_3_ and PO_4_ concentrations, except for scMIT9321, which was affected only by the NO_2_/NO_3_ concentration. scXMU1405 and scXMU1419 showed relatively strong relationships with NO_2_/NO_3_ and PO_4_ concentrations. All subclades in this group were affected by latitude and temperature, except for scMIT9202. In addition, as an outgroup, HLI abundance (normalized to the total abundance of HLI and HLII reads) exhibited a strong negative relationship with temperature and latitude and a relatively weak relationship with photosynthetically active radiation (PAR), indicating that temperature is a key feature that separates HLI and HLII. The results of multiple regression analyses also showed similar results (Table S4).

10.1128/mbio.03027-21.10 TABLE S4 Statistics table for regression models. Download Table S4, XLSX file, 0.03 MB. Copyright © 2022 Yan et al. 2022 Yan et al. https://creativecommons.org/licenses/by/4.0/ This content is distributed under the terms of the Creative Commons Attribution 4.0 International license.

**FIG 6:**
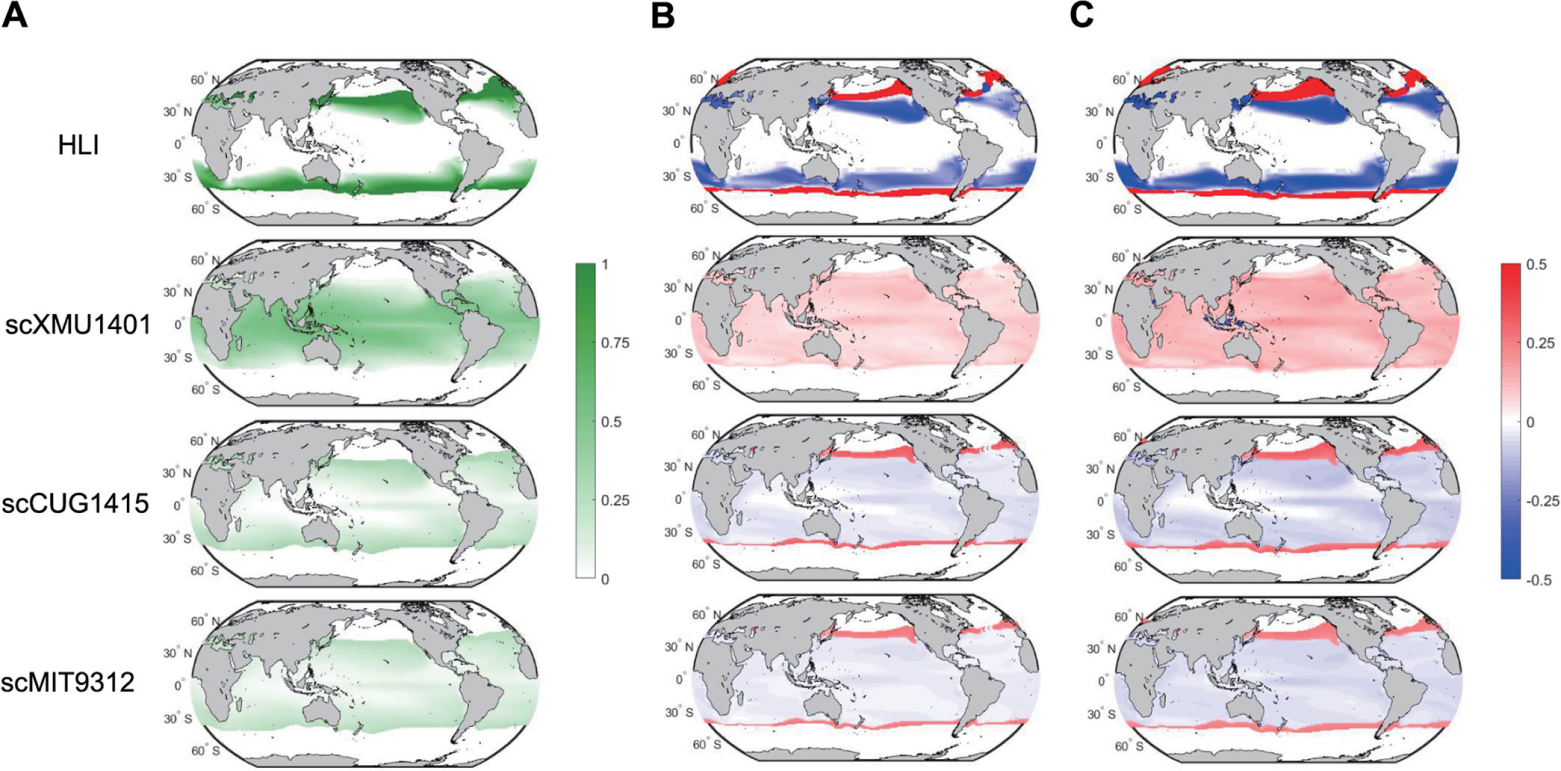
(A) Present global relative abundance and distribution of HLI and major HLII subclades at the sea surface (1990s). Changes in relative abundance between present and future climate (1990s and 2090s) at the sea surface for HLI and major HLII subclades under RCP 4.5 (B) and 8.5 scenarios (C). For statistical analyses, HLI relative abundance was normalized to relative abundance of all HL (HLI and HLII). Relative abundances of HLII subclades were normalized to relative abundance of all HLII.

In this study, temperature was identified as a key factor associated with subclade distributions within HLII, with relatively high explanatory power for the relative abundances of scXMU1401, scCUG1415, and scMIT9312 (Fig. 5). Sea surface temperatures are predicted to increase under future global warming (42, 43). Therefore, we used univariate regression models to predict the relative abundances of HLII subclades and HLI in the global ocean for the 1990s and 2090s with simulated sea surface temperatures of historical and RCP 4.5 and 8.5 scenarios (Fig. S6) (42). HLI and the three HLII subclades for which temperature had relatively high explanatory power were selected for the model predictions (Fig. 5 and Fig. S6). To define the geographic limits of HLII and HLI in the models, the surface ocean temperature boundaries were set to 11°C to 29°C and 14°C to 33°C, respectively, based on the results of metagenomic detection. The results for the model with the present scenario (1990s) showed that HLI was abundantly present in mid-latitude oceans (20° to 45°, either N or S). Within HLII, scXMU1401 (HLII-SG) was the most abundant subclade in low-latitude oceans (30°N to 30°S) (Fig. 6A). The strains scCUG1415 and scMIT9312 in HLII-DG showed a pattern similar to that of HLI but with considerably lower abundance (Fig. 6A). The predicted results for the 2090s showed that the relative abundance of high-temperature-tolerant scXMU1401 (HLII-SG) is likely to increase by 21% and 35% under RCP 4.5 and 8.5, respectively, and its distribution limits will expand toward higher latitudes (Fig. 6B and C). In contrast, the relative abundance of HLI was predicted to decline by 36% and 55% in the 2090s under the RCP 4.5 and 8.5 scenarios, respectively. The predicted changes in HLI relative abundance were uneven at different latitudes, declining sharply at low latitudes (20° to 30°, either N or S) but increasing at higher latitudes (30° to 50°, either N or S), thereby moving its distribution further away from lower latitude oceans (Fig. 6B and C). The predicted patterns for scCUG1415 and scMIT9312 were similar to that of HLI (Fig. 6B and C).

10.1128/mbio.03027-21.6 FIG S6 (A) Decadal average sea surface temperature in 1990s. Change of decadal average sea surface temperature from 1990s to 2090s under RCP 4.5 (B) and 8.5 (C) scenarios. Relative abundance observations for *Prochlorococcus* HLI and major HLII subclades. Relative abundance as a function of temperature for HLI (D), scXMU1401 (E), scCUG1415 (F), and scMIT9312 (G). The lines show the output of linear regression models for temperature. Download FIG S6, PDF file, 0.5 MB. Copyright © 2022 Yan et al. 2022 Yan et al. https://creativecommons.org/licenses/by/4.0/ This content is distributed under the terms of the Creative Commons Attribution 4.0 International license.

## DISCUSSION

*Prochlorococcus* has continuously evolved to adapt to the marine environment, and it can be considered the most recent evolutionary form of cyanobacteria (44). The diverse clades and subclades of *Prochlorococcus* increase its genomic diversity and environmental adaptability, allowing it to become the dominant species in its environment. *Prochlorococcus* HLII is one of the most recently evolved *Prochlorococcus* clades (22). In this study, we report that *Prochlorococcus* HLII consists of three broad subclade groups that can be further divided into ecologically coherent subclades based on their genomic, and ecological differences. Our results suggest that subclade genome differentiation has played an important role in the evolution and ecological niche partitioning of *Prochlorococcus* HLII. After its divergence from the common ancestor of all HLII subclades, HLII-SG may have adapted primarily (e.g., streamlined nonessential genes and increased GC content) to the upper mixed euphotic zone where it was exposed to high temperatures, high photon fluxes, UV radiation, and nutrient limitation. As a result, HLII-SG accounts for the majority of HLII in the upper euphotic zone. In contrast, HLII-DG may have acquired more accessory genes as it gradually adapted to the mid-deep euphotic layers, which feature strong environmental gradients. As a result, their overall genome sizes have increased and their GC contents have decreased. Therefore, gene gain and loss as well as changes in GC content appear to be ongoing and important adaptation strategies of HLII subclades to different environments. Our results also indicate that *Prochlorococcus* HLII has evolved via long-term adaptation to the high-light and high-temperature environment of the upper surface ocean (4, 22, 26); however, its subclades retain the ability and tendency to adapt to different environments, even in the deep euphotic zone. We note that in this study, we use “transition group” (HLII-TG) to refer to several complex HLII subclades and strains with genomic, physiological, and ecological differences. Because HLII-TG appears to contribute to the evolutionary process of HLII-SG, more detailed studies should be conducted for this group in the future.

Rising ocean temperatures will change the productivity and composition of marine phytoplankton communities, affecting global biogeochemical cycles (45). Temperature has been suggested as an important ecological factor that separates HLII from HLI (17, 46). Our results indicate that temperature and nutrient concentrations drive the microdiversity among HLII subclades, and different subclades are associated with different combinations of environmental parameters. Similarly, Larkin et al. (31) found that differences in the relative abundance of HLII haplotypes in the Indian Ocean are driven by nutrient availability and temperature (31). Our model predictions show that, under future sea surface warming, the HLII subclades, especially HLII-SG, will be much more abundant than at present, and their distribution will expand toward mid-latitude regions, while the relative abundance of HLI and HLII-DG will decline sharply in low-latitude areas but their distribution will shift toward higher latitudes. Our results are consistent with those of a previous study that found that rising temperatures this century will cause poleward shifts in species’ thermal niches and a sharp decline in tropical phytoplankton diversity (45). However, our findings represent a much finer-scale phylogenetic level. *Prochlorococcus* absolute abundance is expected to increase by one-third by the end of this century in response to global warming (5). In comparison, our results showed a change in relative abundance of *Prochlorococcus* ecotype and subclade level under global warming. In this study, temperature is found as a key driver controlling the relative abundances of *Prochlorococcus* HLII subclades; however, a few recent studies have shown that ocean warming may also interact with other environmental factors, like nutrients, Fe availability, pCO_2_, and light, and have profound influences on phytoplankton community (47–49). It is necessary to consider the interactions between ocean warming and other primary factors to reveal a comprehensive picture of distributions and abundances of HLII subclades in the future ocean.

Using the strategy of diverse subclade differentiation, *Prochlorococcus* HLII enhances its ability to adapt to different environments while maintaining streamlined single-cell genomes that permit rapid reproduction with low energy and nutrient demands. These characteristics have allowed HLII to become the most abundant ecotype of all *Prochlorococcus* and even all marine phytoplankton. The HLII subclades have a long evolutionary history, and their subclade niche partitioning is complicated and ancient. In this study, we conducted hierarchical clustering analyses at two levels (clade and subclade) to reveal the detailed phylogenetic relationships in HLII. However, our results are still insufficient to clarify the relationships in this huge and complex system. Kashtan et al. (23) found that HLII is composed of hundreds of subpopulations with distinct “genomic backbones.” These closely related HLII subpopulations are estimated to have diverged at least several million years ago based on the comparison of environmental single-cell genomes (23). Fundamental differences in the diversity and genomic structure of HLII exist among different oceans (23, 24). Although many single-cell and metagenomic sequences have been obtained, limited isolates and whole-genome sequences are available, which hampers detailed investigation into the fundamental differences in the diversity and genomic structure of HLII strains. Therefore, more efforts should be made to isolate many new and representative HLII strains for analyses of whole-genome sequences and physiological characteristics. Such analyses may provide a more comprehensive picture of HLII subclade differentiation based on differences in genomic and phenotypic characteristics. Overall, our results shed light on the divergence of *Prochlorococcus* HLII and provide a basis for predicting shifts in distributions of HLII subclades. Our analyses suggest that the expected increase in surface ocean temperature will cause large-scale changes in fine-scale *Prochlorococcus* community structures. Because of the extremely large population size of HLII, these changes are expected to have substantial impacts on ocean ecosystems and global biogeochemical cycles.

## MATERIALS AND METHODS

### Isolation of *Prochlorococcus* HLII strains.

Twelve *Prochlorococcus* HLII strains were isolated from the western Pacific Ocean and the South China Sea in 2014 (see Fig. S1 and Table S1 for details of isolation locations). Seawater was collected with a Niskin bottle and subjected to gravity filtration through double polycarbonate filters (Millipore, USA) with a pore size of 0.6 μm (50, 51). A Pro2 medium nutrient stock solution was then added to the filtrate (50). To avoid cross-contamination, the filtration devices were acid-washed between filtering seawater from different sources. The filtrate was placed into an onboard incubator for initial enrichment. After confirmation by flow cytometry, enriched *Prochlorococcus* were purified using a modified dilution-to-extinction method (52), as described previously (53). The *Prochlorococcus* strains were maintained at a constant temperature of 22°C under a continuous light intensity of 10 to 20 μmol photons m^−2^ s^−1^.

### Genome sequencing, assembly, and annotation.

Genomic DNA was collected from 25-mL laboratory cultures by centrifugation (10,000 × *g*, 30 min) and extracted using the QIAamp DNA minikit (Qiagen, Hilden, Germany). A 1-μg portion of the extracted DNA was fragmented using a Covaris ME220 focused-ultrasonicator (Covaris, Woburn, MA, USA). DNA libraries then were constructed using the NEBNext Ultra DNA library prep kit for Illumina according to the manufacturer’s instructions (NEB, Beverly, MA, USA). Bidirectional sequencing was conducted using 10 ng library DNA on an Illumina HiSeq 4000 instrument with a read length of 150 bp or an Illumina MiSeq instrument with a read length of 250 bp (Table S1). All library construction and sequencing procedures were performed at the Shanghai Hanyu Biotechnology Co. (Shanghai, China). To recover *Prochlorococcus* genomes from nonaxenic cultures, genome assembly was performed as described in our previous study (52) using a binning workflow modified from a well-known method (54). After assembly, all contigs were manually checked and reordered using the “Move Contigs” tool in Mauve v2.4.0 (55). The 12 assembled genome sequences were annotated using tools at the RAST online server (Rapid Annotation using Subsystem Technology; FIGfam version release 70) (56) and the KEGG database (57). For comparison, we also reannotated 21 previously published *Prochlorococcus* HL genomes (19 HLII and 3 HLI) and 19 LL genomes using the same methods (Table S1).

### Phylogenomic analysis of HLII subclades.

Multiple methods were used to construct the evolutionary relationships among *Prochlorococcus* HLII strains. First, ITS sequences were aligned using MAFFT v7 (58) and a phylogenetic tree was constructed using the maximum-likelihood approach with 1,000 bootstrap replicates in RAxML v8.0 (59). Second, phylogenetic trees were constructed using concatenated protein sequences encoded by core genes. In brief, the protein sequences encoded by core genes defined at multiple amino acid similarity levels (ranging from 1,502 genes at 50% to 69 genes at 95%) were concatenated and then aligned with MAFFT v7 (58). The phylogenetic trees were then constructed using a maximum-likelihood approach with 1,000 bootstrap replicates in RAxML v8.0 (59). Finally, phylogenetic trees were constructed using single nucleotide polymorphisms (SNPs) from core genes defined at similarity levels ranging from 50% to 95%. Core genome polymorphic bases were identified and concatenated using the Panseq online server (60) with the following parameters: BLAST fragmentation size, 500 bp; homology fragment similarity percent identity cutoffs, 50 to 95%; BLAST size, 20. SNP-based phylogenomic trees with 1,000 bootstrap replicates were then constructed using RAxML v8.0 (59). All phylogenetic trees presented in this study were visualized using MEGA 7 (61).

### Environmental distribution of HLII subclades.

We searched the TARA Oceans metagenome database by following a reproducible workflow (http://merenlab.org/data/2018_Delmont_and_Eren_Metapangenomics/) that focused on linking *Prochlorococcus* pangenomes and metagenomes (27). In brief, 107 TARA Oceans metagenomes (fractions 0.2 to 1.6 μm and 0.2 to 3 μm in size) from the European Bioinformatics Institute repositories (Table S2) were downloaded and quality filtered. These samples covered low- and middle-latitude surface/subsurface waters (5- to 188-m depth) of major oceans. An anvi’o contig database describing 54 *Prochlorococcus* genomes was generated and mapped to quality-filtered metagenomic short reads from the TARA Oceans samples. To ensure that there was no substantive cross-recruitment from LL clades to HL clades, 20 LL genomes were added to the contig database. To investigate the influence of depth, we separated the 89 TARA Oceans metagenomes in which HL genomes were detected into the surface layer (5-m depth); the 17- to 40-m layer; the 42- to 70-m layer; the 75- to 115-m layer; and the 120- to 188-m layer. To investigate the influence of temperature, we separated the samples into 15 temperature ranges between 0°C and 31°C. Information on the reference genomes and the TARA Oceans metagenomes used in this study is presented in Table S2.

### Pangenome analysis of HLII strains.

Fragment-based pangenome analysis of the 31 HLII strains was performed using the Panseq online server (60) with parameters set as described above. Multiple BLAST fragmentation sizes (500 to 5,000 bp) and homology fragment similarity cutoffs (50 to 70%) were tested. RAST gene functional subsystem analysis was performed (56). Gene-based pangenome analysis was conducted using the Bacterial Pangenome Analysis Pipeline (BPGA) v1.3.0 (62), which uses USEARCH algorithms to identify core genes. Hierarchical clustering based on Euclidean distance was performed using binary data, i.e., the presence or absence of accessory genes in each genome. Two amino acid similarity levels (70% and 50%) were used to identify core genes with significant amino acid variations. The Harvest suite (including Parsnq and Gingr) (34) was used to profile SNP positions across the 31 HLII genomes, with the “random reference,” “recombination detection,” and “inclusion of all genomes” options selected. Two criteria were used to identify core genes with substantial SNP variations: (i) the gene had SNPs in an aligned 100-bp window with <50% identity or (ii) the gene had SNPs in an aligned 100-bp window with >20 indels. The number of gene gains and losses were assessed using Count software (63), which uses a Dollo parsimony method for estimating the ancestral state of every gene in the data set using the core genome SNP tree as the reference. The Dollo parsimony method was used to infer the presence-absence of each gene cluster at each node of the core genome SNP tree and to calculate the number of gene gains and losses on each branch (63).

### Modeling analysis.

Statistical analyses were conducted, resulting in predictive models for the relative abundance of the HLII subclades and HLI at warmer temperatures. Statistical analyses were performed using the R statistical platform (R software, v. 3.6.1; Vienna, Austria). Model prediction and visualization were performed using MATLAB 9.5.0. Environmental factors were gathered from the environmental data set of TARA Oceans (64). To calculate the PAR at different depths, 8-day average diffuse attenuation coefficient at 490 nm (Kd490) values were derived from satellite data (Aqua satellite, 4 km). Detailed information on the environmental factors associated with the TARA Oceans metagenomes used in this study is provided in Table S2.

Correlation analyses were conducted to test for possible autocorrelations among the environmental variables. Univariate regression analyses (linear and quadratic) were used to identify relationships between the measured environmental variables and the relative abundances of the HLII subclades and HLI, using *R*^2^ values of the regression model to evaluate the explanatory power of each environmental variable. The resulting univariate regression models were used to predict the annual average relative abundance of HLII subclades and HLI in the global surface ocean under different temperatures in 1990 to 2000 and 2090 to 2100. Simulated sea surface temperatures under historical and RCP 4.5 and 8.5 scenarios (42) were generated from three global circulation models (CSIRO Mk3 6 0, CanESM2, and HadGEM2 ES) in the Coupled Model Intercomparison Project (https://esgf-node.llnl.gov/search/cmip5/) (43). Multivariate regression analyses were also conducted to identify relationships between the environmental variables and the relative abundances of the HLII subclades and HLI, using Akaike information criterion (AIC) to add or remove variable during the stepwise procedure and *R*^2^ values to evaluate the explanatory power of multiple environmental variables.

### Data availability.

The whole-genome sequence data of the 12 HLII strains reported in this study have been deposited in NCBI GenBank and the Genome Warehouse of the National Genomics Data Center under BioProject numbers PRJNA611498 and PRJCA002394, respectively. Accession numbers for the genomes of individual strains can be found in Table S1.
