# Supplementary figures and images for: Diverse Subclade Differentiation Attributed to the Ubiquity of Prochlorococcus High-Light-Adapted Clade II
Source: mBio. 2022 Mar 14;13(2):e03027-21. doi: 10.1128/mbio.03027-21 (PMC9040837; doi:10.1128/mbio.03027-21)

**A**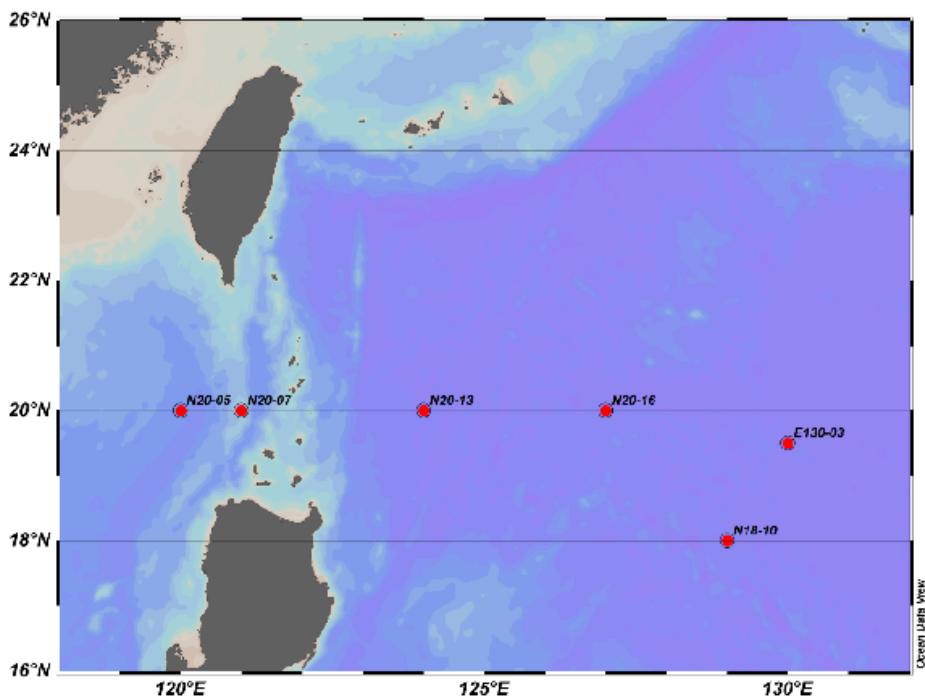**B**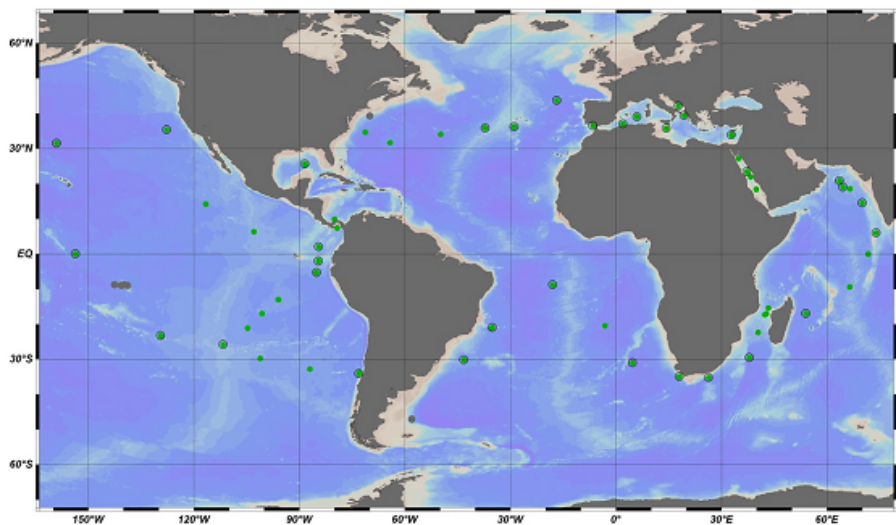

Supplement: FIG S1 [file mbio.03027-21-sf001.pdf]

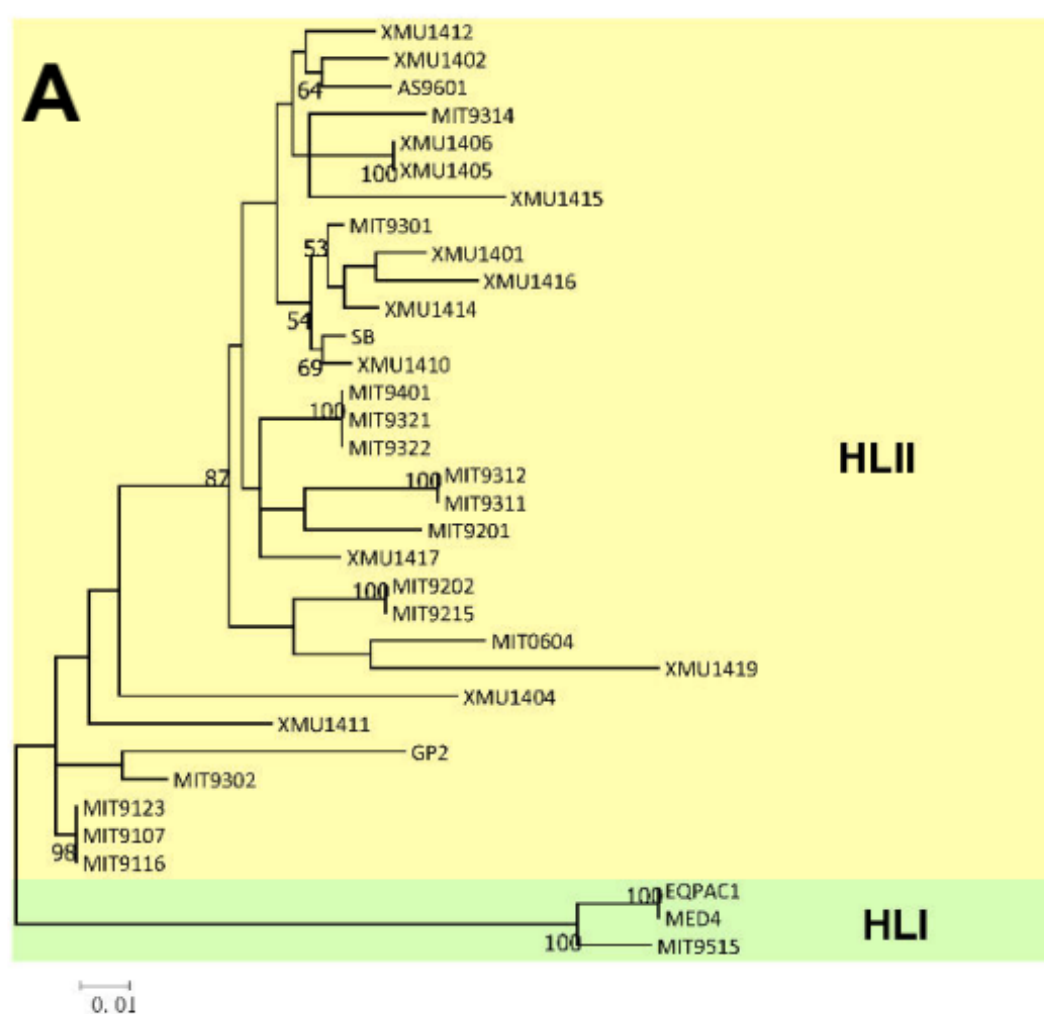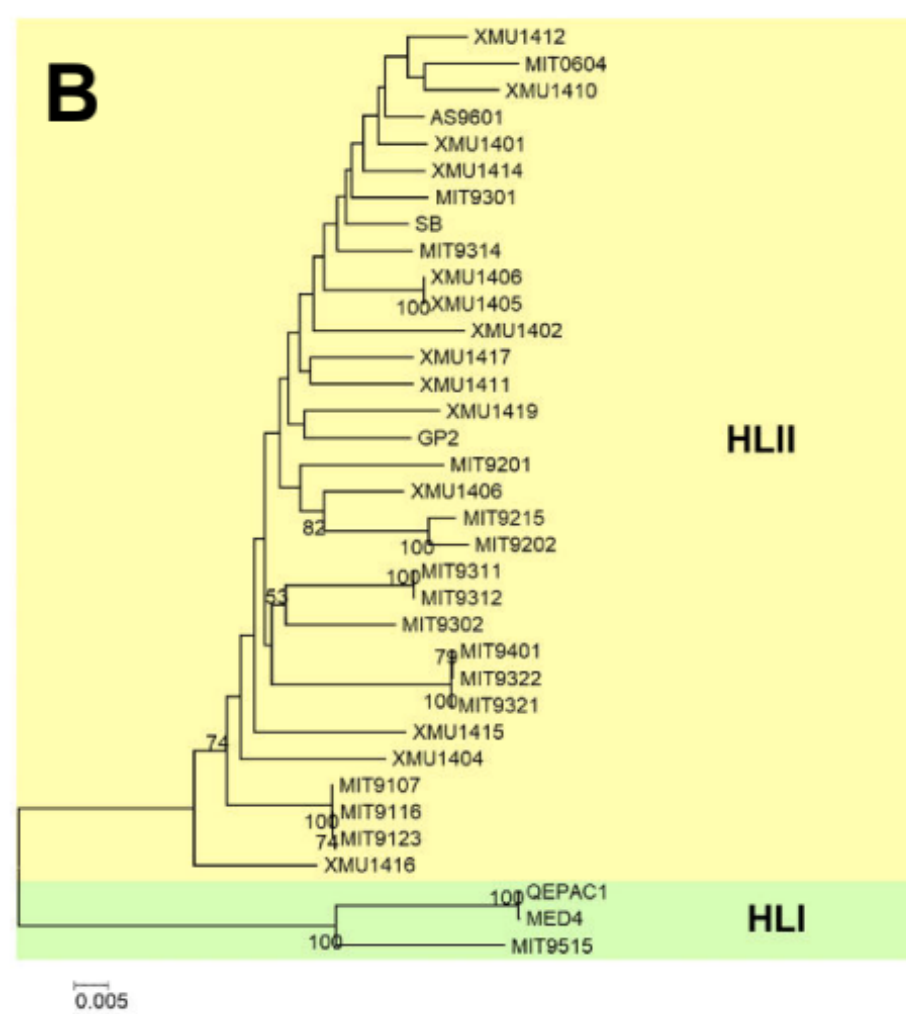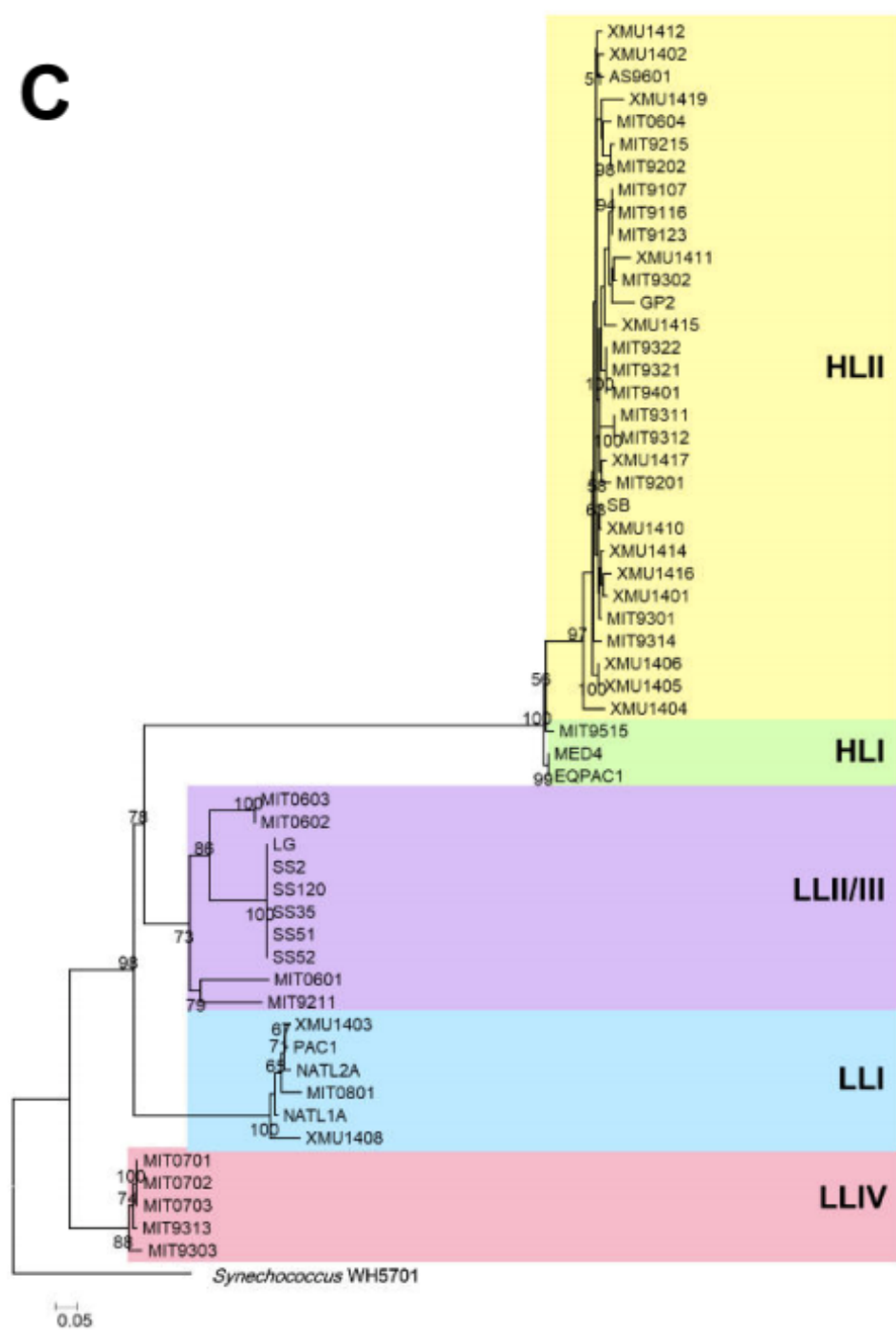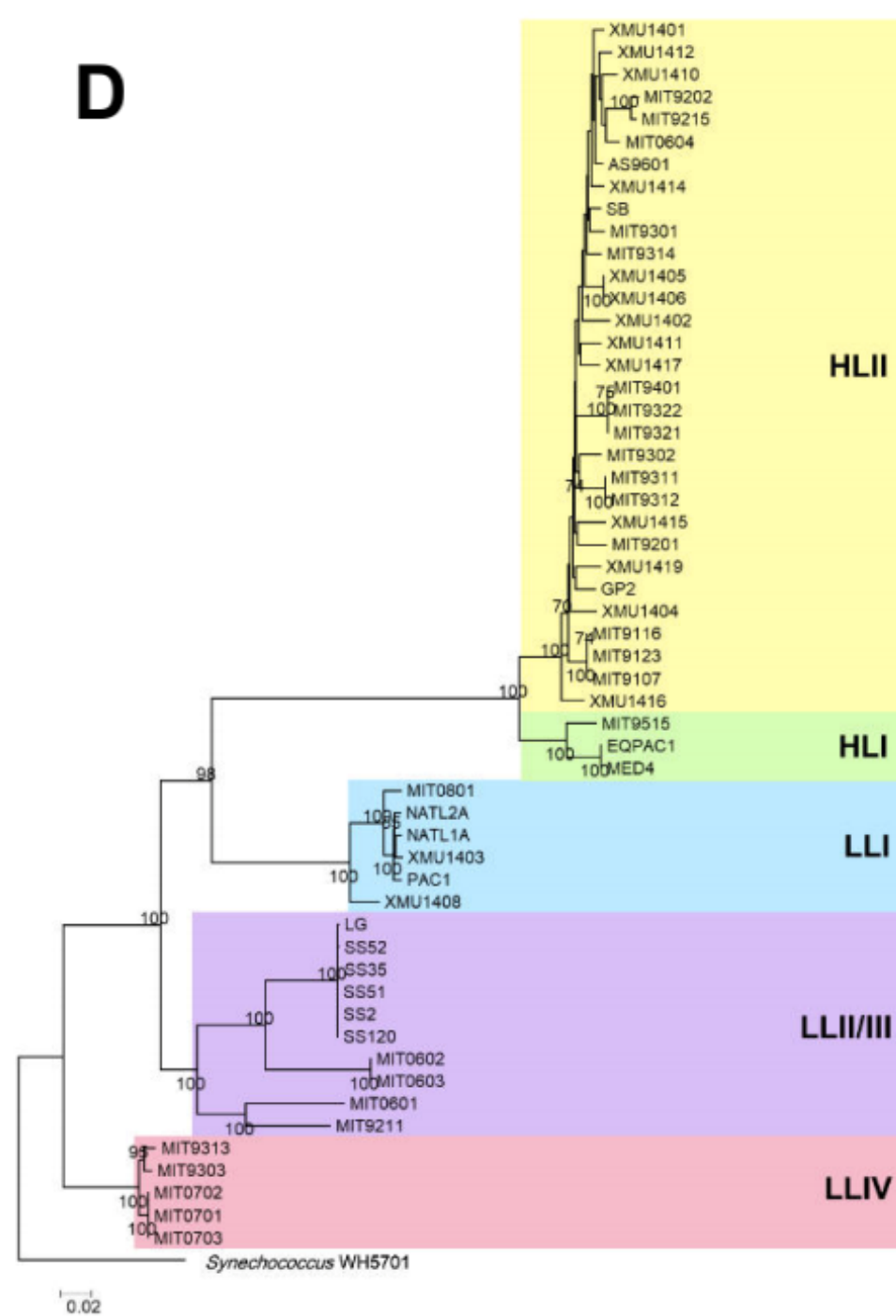

Supplement: FIG S2 [file mbio.03027-21-sf002.pdf]

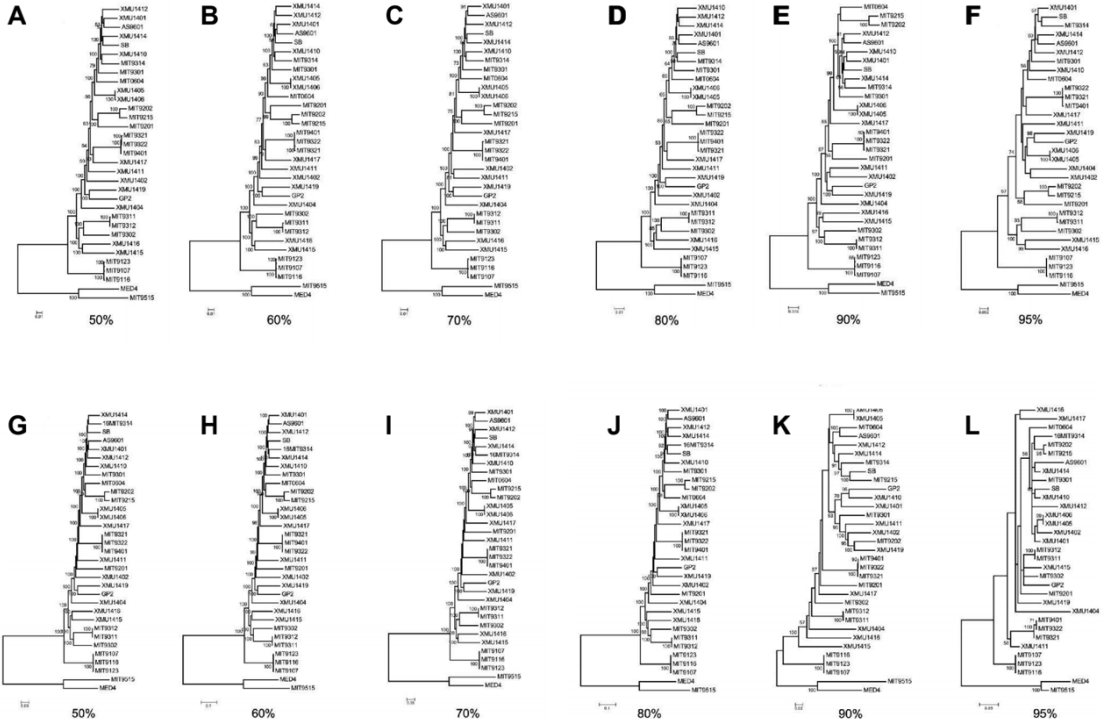

Supplement: FIG S3 [file mbio.03027-21-sf003.pdf]

**A**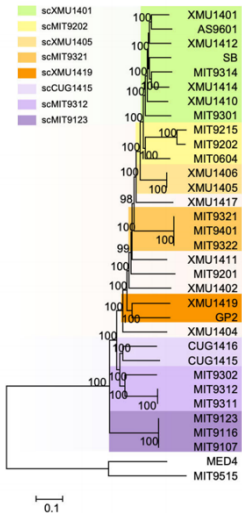**B**

Depth(m)

5 17-40 42-70 75-115 120-188

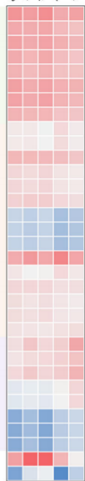

1.0

**C**

Temperature(°C)

13-15 16 17 18 19 20 21 22 23 24 25 26 27 28 29 30

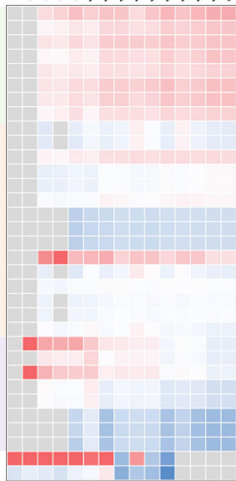

0.5

3.7

SG

TG

DG

**D**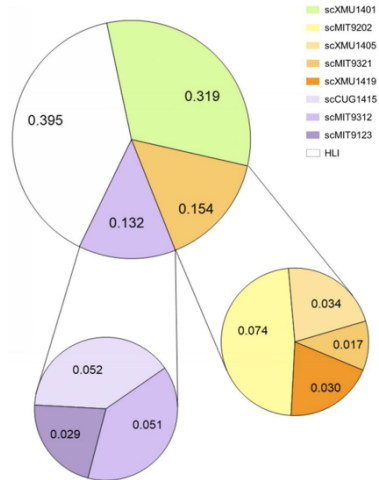

Supplement: FIG S4 [file mbio.03027-21-sf004.pdf]

A

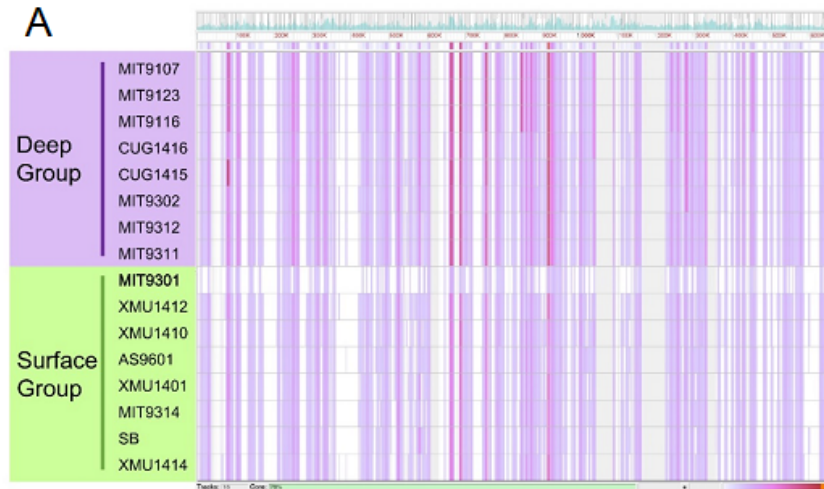

B

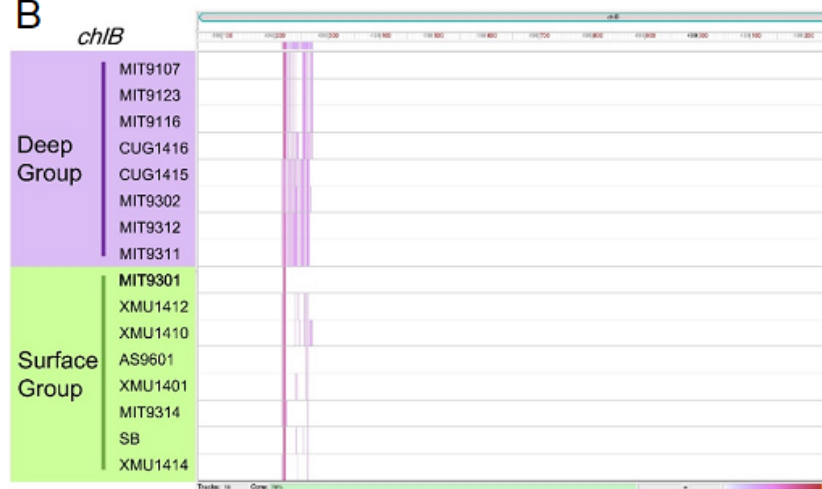

C

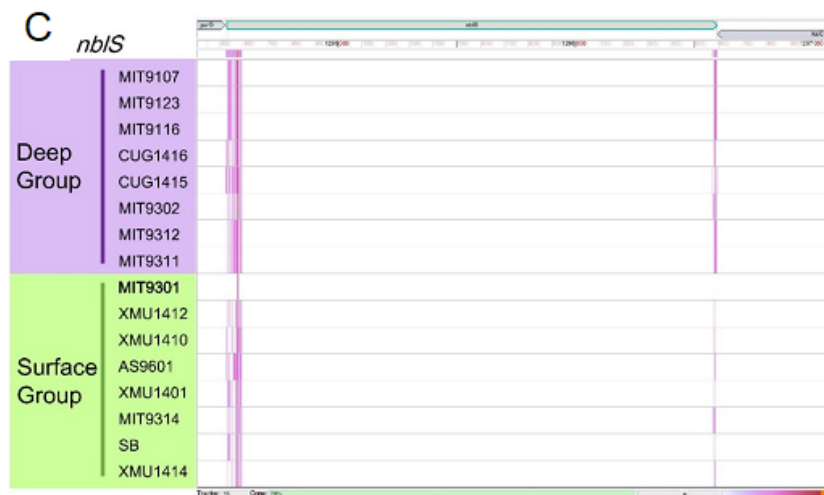

D

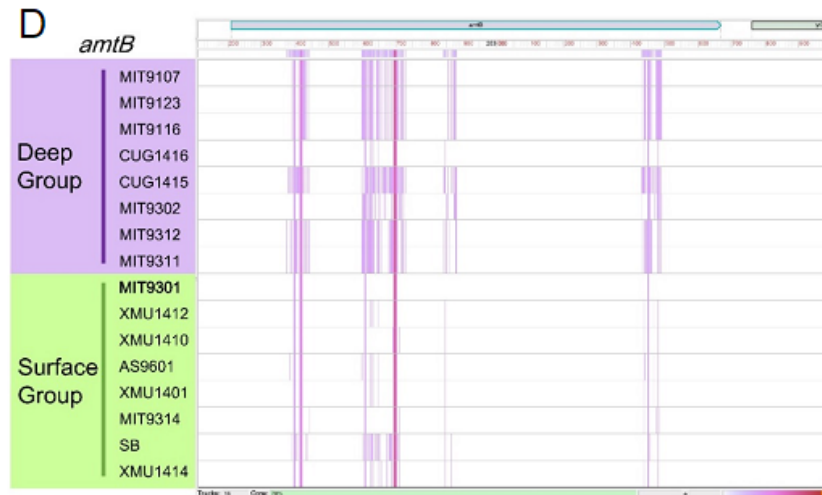

Supplement: FIG S5 [file mbio.03027-21-sf005.pdf]

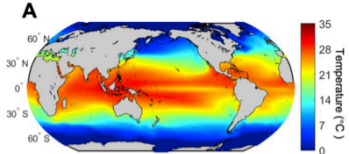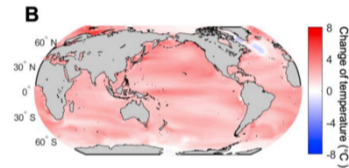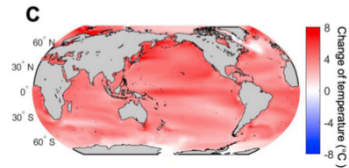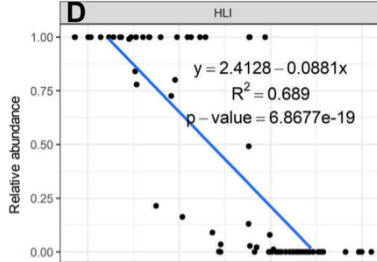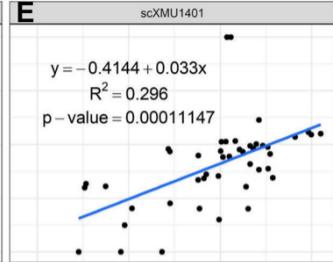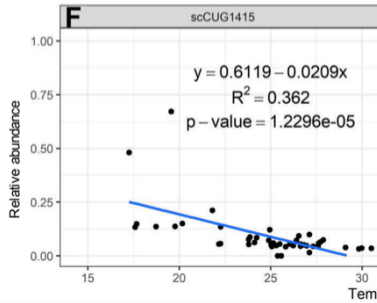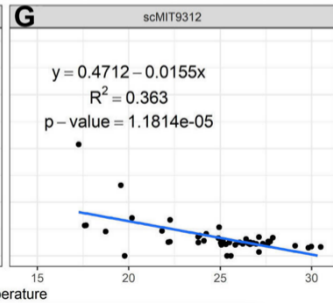

Supplement: FIG S6 [file mbio.03027-21-sf006.pdf]
